# “Grandma, You Should Do It—It’s Cool” Older Adults and the Role of Family Members in Their Acceptance of Technology

**DOI:** 10.3390/ijerph121214999

**Published:** 2015-12-05

**Authors:** Katrien Luijkx, Sebastiaan Peek, Eveline Wouters

**Affiliations:** 1 Tilburg University, School of Social and Behavioral Sciences, Department of Tranzo, P.O. Box 90153, 5000 LE Tilburg, The Netherlands; k.g.luijkx@tilburguniversity.edu; 2 Fontys University of Applied Sciences, Institute of Allied Health Professions, Chair of Health Innovations and Technology, and Tilburg University, School of Social and Behavioral Sciences, Department of Tranzo, Dominee Theodor Fliednerstraat 2, 5631 BN Eindhoven, The Netherlands; 3 Fontys University of Applied Sciences, Institute of Allied Health Professions, Chair of Health Innovations and Technology, Dominee Theodor Fliednerstraat 2, 5631 BN Eindhoven, The Netherlands; e.wouters@fontys.nl

**Keywords:** technology acceptance, technology adoption, family, social network, perspective of older adults

## Abstract

Despite its potential, the acceptance of technology to support the ability to live independently in one’s own home, also called aging in place, is not optimal. Family members may play a key role in technology acceptance by older adults; however, it is not well understood why and how they exert influence. Based on open interviews with 53 community-dwelling older adults, this paper describes the influence of family members, including spouses, on the use of various types of consumer electronics by older adults as was reported by themselves. Such a broad focus enables understanding the use of technology as was reported by older adults, instead of its intended use. Our study reveals that the influence of each family member has its own characteristics. The influence of technology acceptance is a natural and coincidental part of the interaction with spouses and grandchildren in which entertainment and pleasure are prominent. This is also partly true for the influence of children, but their influence also is intentional and driven by concerns. Our study indicates the importance of including all family members when implementing technology in the lives of older adults. Besides information for children about the use(fullness) of devices, it is worthwhile to give grandchildren an important role, because older adults easily adopt their enthusiasm and it might eventually lighten the burden on children.

## 1. Introduction

In general, older adults wish to live independently in their own homes as long as possible, also known as aging in place. In both Eastern and Western countries, older adults cherish their independence because it provides them with the opportunity to live their lives as they always have and to make decisions as has been customary for them [1,2,3,4,5,6]. Governments encourage independent living by older adults because of demographic changes and economic crises. This policy also fits changing perceptions of the position of citizens in need of care and fits the necessity and value to arrange care near older adults, as is the case in the Netherlands [7]. Ensuring sufficient care of adequate quality for community-dwelling older adults will therefore be one of the challenges for the coming years. Technology has the potential to provide a solution for at least part of the care needs of community-dwelling older adults [8,9]. Although various specific electronic devices to support aging in place have been developed (e.g., fall detection systems and monitoring technology), the acceptance of these types of technology, in the sense of (intended) use, as was reported by older adults themselves [10], could probably be greater [11,12,13].

The absence of insight into the experiences, attitudes, and opinions of older adults who use technology to support aging in place might partly explain why such technology is not accepted more often [14,15]. There may be many unintended consequences to using technologies that have not yet been discovered. Older people may be wise in not adopting new technologies. All technology use (and change in general) comes at a cost. Ultimately, individuals make their own decisions about appropriate technology use to suit their needs. In making those decisions, it is plausible that members of the social network influence the acceptance of technology by older adults [16]; currently, it is not well understood why and how they exert this influence. A systematic literature review reveals that, until now, knowledge of the perspective of older adults on technology to support aging in place has been mostly limited to older adults’ intention to use technology sometime in the future, called the pre-implementation stage. Sixteen articles were included, of which twelve focused solely on intention to use technology, three evaluated both intention to use and use, and only one article evaluated use exclusively [17]. Understanding factors that influence the use of technology by older adults in their own homes is important to facilitate the acceptance of technology that could support independent living. Moreover, it is important to study the perspective of older adults themselves, as was done in this paper, because studies reveal that older adults and professional caregivers, including doctors, differ in their perception of what is important to older adults [18,19,20,21].

As was also established by social cognitive theory [22], social relations in general are important to older adults [3,4,6,14,23,24], and previous research indicates that the role of the social network is essential in enabling older adults to use assistive technology as well as household appliances and computers [14,16,17,25,26,27,28]. The influence of children, other family members, friends, and professional caregivers can be both favorable and unfavorable in terms of technology acceptance [17]. For example, female family members often help in selecting and buying technology, while male family members often help with adapting technology to fit the needs of the older adult [14]. On the other hand, older adults and family members do not always agree on the need for technology [16]. Additionally, older adults want to avoid asking too much of other people, especially of their children. In their deliberations of how to keep their autonomy and independence, negative and positive consequences for their children weigh heavily. For example, although most people want to avoid moving to a care facility or nursing home, they are willing to seriously consider it when they think it would be beneficial for their children [3,15,29]. Although members of the social network influence the acceptance of technology by older adults [14,16,17,25,26,27,28]; currently, it is not well understood why and how they exert this influence.

Not all members of the social network seem to be equally important: in comparison with other members of the social network (e.g., friends, peers), family members seem to be the most important when it comes to how older adults live their lives and what solutions they choose when their autonomy is at stake [3,15,29]. Furthermore, it is known that spouses influence each other’s health behavior [30,31,32] and could also be of influence to each other’s acceptance of technology. Therefore, the following research question is central in this paper:

Why and how do family members, including spouses, influence the acceptance of technology by community-dwelling older adults, according to older adults themselves?

In this study, we did not narrow our focus to technology specifically designed to support aging in place but studied various types of consumer electronics. Such a broad focus enables data gathering about technology that is in the home and is used. Studying consumer electronics reveals actual experiences, attitudes, and opinions of community-dwelling older adults instead of expectations in the future. We believe that understanding social factors that influence the acceptance of regular consumer electronics will be helpful in understanding social factors that influence the acceptance of technologies that are specifically designed to assist aging in place. Moreover, many of the regular consumer electronics also have the potential to support aging in place [33,34]. Although both common household technology, like microwaves and electric toothbrushes, and more advanced newer technology, like computer devices and mobile phones, were discussed, this paper elaborates on the latter category for two reasons. First, barriers to buying and using advanced technology are expected to be greater for older adults compared with barriers to accepting regular household technology [33]. These insights can more easily be translated to technology designed to support aging in place. Second, the influence of members of the social network was more obvious when older adults talked about advanced technology.

## 2. Methods 

A qualitative study was conducted among community-dwelling older adults in a town in the south of the Netherlands. The interviews, on which this paper is based, represent the first measurement of a longitudinal qualitative study. Various informants were asked to bring us into contact with persons aged 70 years and older who were living independently. These informants were asked because they had frequent contact with older people in different roles: they worked for a homecare provider, a local senior volunteer organization, or a tablet pilot project. The tablet pilot project, called “Domovisie”, aimed to employ the use of tablets for aging in place by providing older adults with a tablet for one year. The informants were asked to bring us into contact with a varied sampling of people who could be willing to talk about technology in daily life: we aimed to include both women and men, people living alone as well as those living with a partner, and people both with and without a need for care and support. Furthermore, we asked them to only select people who were competent in the Dutch language and who were capable of being interviewed unhampered by cognitive or other problems. To further increase the sample, also included were some people we met at the local shopping center and others we met through the respondents themselves.

In the second half of 2012, 72 potential respondents were informed by letter about the study and invited to participate. Approximately one week after receipt of the letter, the interviewers contacted the potential respondents by phone to answer possible questions, ask for their cooperation, and, if appropriate, make an appointment for the interview. The interview team consisted of two interviewers, psychologists trained in interview techniques, and two observers, university lecturers with a background in health care. Each respondent was visited at home by an interviewer accompanied by an observer. If a household consisted of more than one person, only one of them participated in the study. In some cases, the spouse was present and, although the interviewers directed their questions to the respondent, spouses sometimes clarified or added insights to the interview. At the beginning of the visit, which lasted between one and a half and two hours, the informed consent form was discussed and signed by the respondent. Subsequently, the respondent was asked whether he/she in the last year had experienced life events that were meaningful to them, using the Social Readjustment Rating Scale (SRRS) [35] adjusted for older adults and to fill in the Tilburg Frailty Indicator (TFI) [36] and the Mini-Mental State Examination (MMSE) [37] to get an impression of both physical and cognitive health status as well as major life events. Although this was never the case in practice, the interview protocol instructed that when a participant scored lower than 24 on the MMSE the interview needed to end [38]. The next step was a tour of the house, in which an inventory was made of the technology present. For each device, frequency and type of use was recorded. After these preparatory activities, a maximum of three devices was selected, based on preferences of participants who sometimes displayed strong feelings, both positive and negative, towards certain devices and on suggestion by the researchers who aimed for variation in devices that were and that were not integrated in the daily lives of older adults. Respondents were interviewed to learn how these specific devices originally came into their home and to understand what influenced both use and non-use. For each device, we asked in an open way about factors that could be of influence. In most interviews, the role of family members, especially of spouses, children and grandchildren, naturally came to the fore. When that was not the case, but there were indications of the influence of the social network, we asked the respondent to further elaborate on that influence.

Furthermore, the topic list was based on a systematic review of factors that influence acceptance of technology for aging in place [17].

With permission of the respondent, the interview was digitally audio recorded and transcribed verbatim. In total, five researchers were involved in the coding of the interviews. Each transcript has been coded independently by two of these researchers who discussed their coding to reach consensus. Factors described in the systematic literature review [17] that inspired the topic list, were initially used as sensitizing concepts. Discussions in the coding pairs and in the whole coding team lead to the introduction of new codes, based on what respondents had told in the interviews. Coding and analyses of the interviews were aimed at understanding influencing factors for technology acceptance, including the effect of the social network. To describe how and why family members influence the acceptance of technology by older adults, we focused on the analysis on codes referring to the role of the social network. Inspired by grounded theory, our analysis was inductive [39]. Analyzing the relevant codes and associated text fragments several times, we discovered that spouses, children and grandchildren have their own reasons for and ways of influencing the purchase and use of specific devices. The reasons and ways of influence were leading in our analysis. We elaborated on those devices that had the potential to learn us about the reasons and ways of influence.

The study protocol of the entire longitudinal qualitative study was approved by the Psychological Ethical Commission (PETC) of the Tilburg School of Social and Behavioral Sciences at Tilburg University (EC-2012.04).

## 3. Results

Of the 72 potential respondents who were invited to participate in the study, 53 gave their consent, a response rate of 73.6%. At the beginning of three interviews, it was discovered that these respondents were younger than 70 years: two were 69 years old and one was 68 years old. It was decided to include these interviews because their stories fit easily with the stories of our other respondents, and since a considerable drop-out rate in subsequent measurement moments is not unusual, as many respondents as possible had to be included in the first period of data gathering.

Table 1 shows some general characteristics and health indicators of our respondents. Their mean age was 78 years, and 43 (81.1%) of them were between 70 and 85 years. Most (64.2%) were female, which more or less corresponds to the gender distribution in the older Dutch population: 55% of the Dutch population over 65 and 70% of the population over 85 is female [40]. Furthermore, our respondents had relatively little education and the majority lived alone. All respondents reported having one or more chronic conditions. Most often were mentioned: high blood pressure (49.1%), arthritis in hips or knees (47.2%) and severe or persistent pain or limitation in the back (41.5%). One in five experienced mild cognitive problems according to the Mini Mental State Examination (MMSE) and more than half were frail according to the Tilburg Frailty Indicator (TFI), which is similar to the occurrence of frailty in the Dutch population [41].

All respondents, with the exception of two, described the role of members of their social network when talking about technology in their homes and lives. Although spouses were also important, our respondents told us more frequently about the influence of children and grandchildren. For the purposes of this study, the term “children” includes both the respondents’ own children and their children’s spouses. Of the 51 respondents who mentioned the role of members of the social network, 11 referred to spouses, while 29 referred to children and 15 to grandchildren.

In the following sections, the influence of the spouses, children, and grandchildren on technology acceptance will be elaborated on. All of these network members appear to have their own ways and reasons to influence respondents’ use of computer devices (computers, laptops, and tablets). Therefore, this type of technology is detailed in each of the following sections. The use of other types of technology will be discussed when the role of a specific type of family member became evident. As such, electric bikes, mobile phones, and personal alarms are elaborated on.

**Table 1:** General characteristics and health indicators (n = 53).

Age (Mean 78)	N	%
65–69	3	5.7
70–74	11	20.8
75–79	21	39.6
80–84	11	20.8
85–89	5	9.4
90+	2	3.8
**Gender**		
Female	34	64.2
Male	19	35.8
**Education**		
None or primary education	17	32.1
Pre-vocational education	11	20.8
Secondary vocational education	5	9.4
Secondary education	13	24.5
Higher education	7	13.2
**Living arrangement**		
With partner	15	28.3
Alone	38	71.7
**Number of chronic conditions**		
0–1	1	1.9
2–3	20	37.7
4–6	20	37.7
7+	12	22.6
**Cognitive capabilities according to MMSE**		
No cognitive problems (MMSE: 27–30)	42	79.2
Some cognitive problems (MMSE: 24–26)	11	20.8
**Frailty according to TFI**		
Not frail (TFI ≤ 4)	25	47.2
Frail (TFI ≥ 5)	28	52.8

In general, the influence of both spouses and grandchildren is a natural and coincidental part of their interaction. When one spouse buys and uses a device, the other naturally comes in contact with it. Older adults easily adopt the enthusiasm of their grandchildren for technology, especially computer devices. This also holds true for children. Their influence, however, is also driven by concerns about the well-being of the older adult and is therefore more intentional.

### 3.1. Spouses: Natural and Coincidental 

Although fifteen of our respondents lived with their spouses at the time of the interview, eleven explicitly said something about the influence of their spouses on the acceptance of technology. It should be noted that of these eleven, six (two women and four men) were living with their spouses at the time of the interview while the spouses of the other five had passed away. In some cases, the spouses were present during the interview and clarified or added some insights to the conversation. In general, the influence of spouses was rather coincidental; it just happened to be part of their natural interaction when it came to the acceptance of computer devices, electric bikes, and mobile phones. Most spouses supported each other in the acceptance of technology and also exerted intentional influence; some spouses suggested that their partners buy an electric bike because they were convinced it would be better for them. In a few cases, husbands were not supportive of computer use of their spouses.

#### 3.1.1. Computer Devices

The use of computer devices was mostly limited to surfing the Internet, video telephony, and electronic banking. One woman tried Internet dating, but she did not really like it. In general, our respondents preferred a laptop or tablet over a desktop computer placed in a separate room because they preferred to be together when one of them used a computer device. However, at the same time, they found a personal computer in the living room was too intrusive. The purchase of a computer device often was initiated by just one of the spouses. Although the other spouse originally did not display a manifest interest in this type of technology, he or she would come in touch with a computer device and the Internet by coincidence when this type of technology entered the home. Among our respondents, women were more often the initiator to buy a computer compared with men. Four of the men we interviewed told us that their wives were the reason they owned a computer. A widower who did not have any interest in computer devices at all replied to our question why he had accepted the computer that was given to him and his wife when she was still alive:

For my wife…otherwise that computer would never have come here.

*(Male, 77 years, living alone)*

Despite his indifference, which was already the case when he had to use a computer at work, he now and then played a game on the computer. Another male respondent followed his children and grandchildren on Facebook, but left the use of the laptop largely to his wife.

My wife searches the Internet for information about health. I don’t.

*(Male, 73 years, living with wife)*

When explaining their considerations for joining the tablet pilot project “Domovisie” one man said:

In the beginning, when I heard of it, I thought, “That could be something for my wife”.

*(Male, 85 years, living with wife)*

Spouses were not always encouraging and could also complicate the purchase and use of a computer device. One woman, for example, was considering to get her own website to show the art she made, because frequently people asked for her website and she expected to be asked for expositions more often. However, her husband was not in favor; he wondered who knew his wife and would search the Internet for information about her and her work.

In other cases, spouses were a little bit more curious, but in the end did not really use the computer devices. When we asked one of our male respondents if he ever did anything with the tablet that his life partner, who had recently passed away, had purchased, he said:

I solely had a look to know how it works and what it is.

*(Male, 68 years, living alone)*

Later in the interview he said:

I do not use that thing [tablet]. That little device has just entered the home and she has learned to use it, I did not interfere with it.

*(Male, 68 years, living alone)*

#### 3.1.2. Electric Bike

Seven of our respondents told us about their considerations to buy an electric bike; six of them owned one. Three married men and one widowed woman spoke of the influence of their spouses in buying and using such a bike. For respondents who liked to cycle, an electric bike was appealing because it lightened this enjoyable activity that could be physically quite fatiguing, particularly when the people they cycled with had one. None of the respondents who owned an electric bike had been unable to cycle before purchasing one. Our respondents told us that their electric bike helped them to be active and healthy and enhanced daily activities like grocery shopping and visiting family or friends.

One woman bought such a bike because her husband, who was so ill that he was lying in bed in the living room, insisted and argued that it would be better for her knees. As a result, she enthusiastically used the electric bicycle to visit her family and also just enjoyed riding the bike for its own sake.

When the spouse already owned an electric bike, it was natural to purchase such a bike oneself, because it was rather physically demanding to cycle with someone riding a bike with an electric motor when riding a regular bike oneself. Two respondents mentioned this to explain why spouses of older people with such a bike also decided to buy one, sometimes strongly encouraged by their spouse.

And certainly when we cycle together, then it is certainly enjoyable. Because she has a speedy tempo and, on a regular bike, I have to pedal intensively to keep up with her. I had just bought a new regular bike, half a year ago. She said to me, “Come on, please buy an electric bike”. And then I thought, “Yes, it would be nice to also have an electric bike”. Now, I would not want to miss it anymore.

* (Male, 74 years, living with wife)*

After a fall and three to four months of recovery, another respondent did not expect to be able to drive a car or cycle once again. When he was able to cycle again, he wanted to buy a new bike and his wife gave the following advice:

My wife said, “Since you grow older, it is better to buy an electric bike”.

* (Male, 81 years, living with wife)*

#### 3.1.3. Mobile Phone

Many of our respondents owned a mobile phone, but rarely used it. It provided them with the certainty to be able to call someone or to be called when necessary, wherever they were. For many of our respondents, the use of a regular mobile phone, not a smart phone, was rather challenging. One of our respondents, for example, went back to the store because his phone did not work anymore and discovered that a mobile phone needs recharging. Sometimes each of the partners owned and used a mobile phone; more often, they owned one together and one of the spouses was more skilled in its use. Although one of our female respondents used their mobile phone to call her family, her husband always keyed in the number. He said:

You can’t say that you use it yourself.

*(Husband of female, 78 years)*

### 3.2. Children: Natural and Intentional

The narratives of the 29 respondents that elaborated on how children influenced their use of technology learned that this influence was multifaceted. In many cases, children helped their parents purchase and use certain technology. This natural influence was often accompanied by a more intentional influence. Out of concern, children aimed to convince their parents to use certain technology like a mobile phone or a personal alarm. Many of our respondents understood their children’s worries and were therefore also willing to accept technology that they initially did not prefer, while others had the feeling that their children forced their ideas upon them.

The role of children in technology acceptance was most obvious for computer devices, mobile phones, and personal alarms. In elaborating on these devices, we do not differentiate between children and the children’s spouses because in providing advice or support about technology the expertise and experience someone was more important than the presence of a blood tie.

#### 3.2.1. Computer Devices

Children influenced the acceptance of computer devices in various ways. Thanks to their children, older adults got to know the possibilities of computer devices, which could give rise to the intention to purchase one. Often, children facilitated it, sometimes they advised against it, and sometimes our respondents felt pushed by their children to buy a computer. When respondents became aware of an appealing function of a computer device, they were willing to give it a try. For example, respondents who originally wanted to avoid computer devices in their homes, changed their minds when children or grandchildren who lived far away used video telephony to stay in touch.

Seeing their children and grandchildren using a specific computer device triggered the wish in older adults to own this kind of technology themselves. One respondent literally said (with a laugh):

When my children have something, I have to have it also. No, that is just a joke. … I saw it with my children, the tablet, even my grandson who is three years old works with it—he is my great-grandson. When you see it, you think “That is handy”. It is light enough to take it with you. I think it is the invention of the century”.

*(Female, 83 years, living alone)*

For some respondents, the wish to go along with current developments and to not lose connection with society was a reason to buy a computer device. Usually, children encouraged their parents, but we also noticed that some older adults felt pressed by their children, while other children advised against it. One woman answered our question why she and her husband had bought a computer as follows:

My son insisted on it. My husband was not in favor of it, and I was totally not in favor, but all right, you cannot do without it anymore, you have to.

*(Female, 78 years, living alone)*

However, it could also work the other way around. Although the daughter of the following woman was against it, the woman went to the store with her son-in-law to buy a tablet:

My daughter said, “Mum, don’t start with it. A tablet—what do you want to do with it?” I said, “Well, I don’t know”. Then my son-in-law said, “If your mother wants a tablet, I will join her to the store to buy one”.

*(Female, 77 years, living alone)*

Many of our respondents found it difficult to (learn to) use a computer device. Their children were much handier with them, and most of our respondents asked them for help when necessary.

When my computer does not work, my son comes and he fixes it.

*(Male, 77 years, living alone)*

Sometimes, help was provided via telephone, as one woman explained:

When I wonder, “How does it work again?”, then I call them. Yesterday, a very simple thing: the font size was too small. They told me to tick a specific box. And then I think, “Yes, I knew it, but I just lost it for an instant”.

*(Male, 85 years, living with wife)*

A few of our respondents told us about their reluctance to ask too much of their children because they did not want to trouble them. When we asked one of our female respondents if she could ask her sons for help, she answered:

No, they all live too far away—that is not an option. And my daughter is more nearby, but she also knows little about it. Yes, her husband knows, but I do not want that. I do not want to trouble them.

*(Female, 81 years, living alone)*

#### 3.2.2. Mobile Phone

Of the 22 respondents who discussed having a mobile phone in their lives, eight spoke about the influence of their children on accepting the mobile phone. For the children of four of our respondents, it is a reassurance when they had a mobile phone and brought it with them when leaving their home. Although they felt somewhat pressed, they understood the concerns of their children; reassuring the children was enough reason to accept a mobile phone. This acceptance seemed to be incomplete; they owned a mobile phone and carried it with them, but rarely used it. Children also kindly stimulated the acceptance of a mobile phone by purchasing it together or giving advice. In general, our respondents carried a mobile phone to be sure they could call for help if needed.

For one of our respondents, the worries of his daughter about him cycling without carrying a mobile phone was the reason to buy one. He carried it and recharged it but at the time of our interview had never used it. In addition, a serious life event could directly cause the children to insist that their parent(s) buy a mobile phone. One of our respondents told us:

After the first CVA [cerebrovascular accident], my daughter said, “Mum, you have to buy a mobile phone”. Then she gave me one. The children told me to take it with me when I am outside with my mobility scooter.

*(Female, 77 years, living alone)*

Although her children were rather forceful, she agreed with them because she knew her mobility scooter could break down. Another woman was very short about the reason why she owned a mobile phone:

My son instructed me to do so.

*(Female, 78 years, living with husband)*

Many children gave their parent(s) a mobile phone as a present. Some older persons appreciated this gift while others felt pressured and were not really happy with it.

#### 3.2.3. Personal Alarm

Contrary to the other technologies examined, a personal alarm only has a prevention and care function and no other appealing uses. Several respondents who did not have a personal alarm mentioned that they would feel really old when in need of a personal alarm. Respondents who did have a personal alarm stressed the feeling of safety; they were certain that they could call for help when needed.

Ten of our respondents spoke about the personal alarm that they used. A serious life event, such as a fall or the death of the spouse, combined with worries of their children gave people enough reason to accept a personal alarm. Some older adults felt somewhat pressed by their children, but they mostly acknowledged that they themselves also felt safer as a result. One respondent answered as follows when she was asked how necessary the personal alarm was for her:

Well, because my children insisted. I think, it is to reassure them.

*(Female, 80 years, living alone)*

### 3.3. Grandchildren: Natural and Coincidental with Pride and Joy

Fifteen older adults addressed the influence of grandchildren. This was most apparent for computer devices, probably because the gap in knowledge and skills between our respondents and their grandchildren was largest in this domain. The enthusiasm of grandchildren for computer devices and applications was very prominent. Grandchildren were a trigger to buy a computer device and to use specific applications like video telephony and social media. Furthermore, grandchildren were a natural source of support. Older adults displayed pride and pleasure when talking about the abilities of their grandchildren.

#### 3.3.1. Computer Devices

Due to the enthusiasm of their grandchildren for computer technology, older adults were willing, maybe even eager, to buy and use a computer device. Referring to the decision to participate in the tablet pilot project “Domovisie”, a woman said:

The grandchildren also said, “Grandma, that’s great! You should do it, we will help you” and I thought, “Yeah, why not? What could stop me?”

*(Female, 79 years, living alone)*

The following quotation shows that the enthusiasm of the granddaughter about a tablet was a stronger facilitator for this respondent than the skepticism of the daughter was a barrier. However, although this respondent referred to this kind of technology as “nonsense”, she decided to participate in the tablet pilot project “Domovisie”:

My granddaughter says, “Grandma, you should do it—it’s cool”. But my daughter says, “Ma, you won’t understand it; it is of no use to you”.

*(Female, 77 years, living alone)*

Computer devices provided opportunities to interact with grandchildren via, for example, video telephony applications or social media. In particular, grandchildren living abroad were a reason to buy a webcam and use video telephony. For several respondents, interaction with the grandchildren was the most important reason to have a computer and use social media. The following quotation illustrates this importance; even when people did not really like or were not really interested in social media, they used it to stay informed about the lives of their grandchildren.

R: In certain things we are not interested.

Interviewer: Can you give an example?

R: The communication via Facebook and via Twitter. I have an account for my grandchildren; then I see something of their lives. But I do not post messages myself.

*(Male, 73 years, living with wife)*

Grandchildren were also willing to facilitate the use of computer devices; they demonstrated the possibilities (e.g., certain games), installed applications, and helped when necessary. Although they were often too fast when showing how to do something, respondents appreciated this support very much and were hardly reluctant to accept help from their grandchildren. Having the opportunity to ask grandchildren for help provided respondents with comfort and pride, as illustrated in the following citation of a grandfather:

My grandson, he is eleven, will support me. He really knows how to use it. He knows how to search for apps. He says, “Look grandpa, these are for free and for these you have to pay”, and then he has a whole list. Yeah, he’s good at this. I like it very much.

*(Male, 76 years, living with wife)*

The grandchildren of the following respondent taught her much about the use of her computer. They are an anchor for her and she proudly tells about it:

I only have to call him to get him in for help. My other grandson is younger, but he might be even more clever with the computer.

*(Female, 69 years, living alone)*

## 4. Discussion

In answering our research question—“Why and how do family members, including spouses, influence the acceptance of technology by community-dwelling older adults, according to older adults themselves?”—an addition can be made to the scarce body of literature about the perspective of older adults regarding the influence of family members on technology acceptance. Our results show that the acceptance of technology by older adults, in the sense of purchasing and using devices, is not an individual matter; it is influenced by spouses, children and grandchildren, as was earlier established in the Netherlands [16], also in other parts of the world [42,43].

Each category of family members has its own reasons for and methods of influencing. Interest in, purchase of, and use of technology by older adults is influenced by both spouses and grandchildren as a natural and coincidental part of their interaction. When they interact, they also coincidentally see each other’s devices, talk about the devices’ possibilities, and can try them. This is in line with more general theories that suggest that adoption of new behavior and/or technologies is facilitated when individuals can see and try it [22,44]. Often, this nourishes the interest of older adults in technology. Although interactions with spouses are more frequent than with grandchildren, pleasure and entertainment are important facilitators when it comes to influencing technology acceptance in both these relationships. However, the interaction between spouses sometimes also has some intentional aspects.

In general, but not always, spouses are supportive of each other: they persuade or stimulate and help each other in buying or using a specific technology. They naturally come into contact with each other’s devices and may become convinced of the usefulness of it. Furthermore, they are a natural, and often available, facilitator and source of support. Spouses ride their electric bikes together. Sometimes, they even complement each other when using certain devices; for example, one man keyed in the number when his wife wanted to call someone with their mobile phone. The question remains of what happens when one of the spouses dies: Will the other then still ride the electric bike and use the mobile phone, or would that be too much of a challenge? Our results indicate that after the decease of the spouse older adults might not always continue using devices that their spouse brought into the home, but sometimes they do. Therefore, it would be interesting to further study this in future longitudinal research.

Spouses and grandchildren influence technology acceptance of older adults in a rather unintentional way; it is a natural and coincidental part of their regular interaction. This holds true for the influence of children as well, but their influence also has more intentional aspects and is partly driven by concerns. For example, mostly out of concern, children strongly advise their parents to use a mobile phone. Sometimes, a mobile phone is given as a present, which is appreciated by some, while others feel that it is forced upon them. Older adults are rather submissive when they can diminish their children’s worries by adapting to certain technology like a mobile phone or even a personal alarm. Especially when it concerns technology that is not specifically designed for older adults, like a mobile phone, they follow their instructions and rarely go their own way in buying or using technology when their perspective deviates from that of their children. This is more complex when it concerns devices specifically designed for older adults, like a personal alarm, that might have a stigmatizing effect. In those cases, older adults weighed the tradeoff between personal feelings of safety and worries of their children and the possible stigmatization. Many of our respondents have assumed an adaptive management strategy to cope with needing help. Characteristic of this strategy is the adaptation to others’ views about what is necessary. Positive or negative feelings are invoked by the matching of the arranged support and the needs as experienced by older adults themselves [2].

**Table 2:** Summary of results.

**Older Adult—Spouse: Natural and Coincidental**
Both advise each other on what to (not) use
Both can initiate purchase
Both can help each other in using technology
Use by older adult may lead to use by spouse, and vice versa
Together they form an implicit or explicit agreement on who uses what
**Older Adult—Children: Natural and Intentional**
Children advise and help older adults, typically not the other way around
Use by children may lead to use by older adults, typically not the other way around
Children either help older adults in buying technology, or they buy it for them
Children may be inclined to push their parents to use technology, out of concern
Older adults may be inclined to use technology for the sake of their children
Older adults may be inclined to not put a burden on their children
**Older Adult—Grandchildren: Natural and Coincidental with Pride and Joy**
Grandchildren advise and help older adults, typically not the other way around
Use by grandchildren may lead to use by older adults, typically not the other way around
Grandchildren influence older adults by their enthusiasm
Older adults are typically not reluctant to ask their grandchildren for help
Older adults are proud of their grandchildren’s technology related skills

In general, older adults are reluctant to ask too much of their children. Other research suggests a delicate trade-off between maintaining independence and following the opinion of their children. Older adults do this , also because they want to avoid burdening their children [15,29]. Additional studies have revealed the importance of social relationships for older adults and the reluctance to burden their children [3,4,14,24,45]. We also found such indications but can add that older people hardly are reluctant to ask their grandchildren for help. The enthusiasm and help of grandchildren is a clear facilitator in the acceptance of technology by older adults. Filled with pride, older adults tell about the enthusiasm and the abilities of their grandchildren in using computer devices and about their willingness to help when problems arise. Facilitating communication and being informed about the lives of family members, especially grandchildren, is the main reason for older adults to use video telephony or have an account on social media. The results of our study are summarized in Table 2.

When comparing our findings to existing technology acceptance models, it becomes apparent that current models offer a limited take on how and why family members influence older adults’ technology use. For example, the most dominant model—the Technology Acceptance Model (TAM)—only incorporates one social variable: subjective norm (*i.e.*, the degree to which an individual perceives that important others believe he or she should use the new system) [10]. This limitation of TAM was put forward by the original author [10] and is confirmed by our study, which shows that other types of social influence besides subjective norm play a role in the acceptance of technology by older adults, such as the help and support offered by family members. The notion that support may facilitate use is acknowledged by the Unified Theory of Acceptance and Use of Technology (UTAUT) [46,47], which are generally considered to be the successor to TAM. UTAUT incorporates facilitating conditions, which are defined as the degree to which an individual believes that an organizational and technical infrastructure exists to support use of the system [46,47]. However, our study shows that the mere belief that support is available is not enough to facilitate use by older adults; older adults also need to be willing to call upon their relatives to help them.

It is important to note that several authors have attempted to extend TAM, in order to form models that are more suitable to the context of older adults. One example is the Senior Technology Acceptance Model (STAM), as described by Renaud and van Biljon [48]. This model, which is aimed at predicting acceptance of mobile phones by older adults, also entails social influence. According to the authors, their concept of social influence aligns with a variable which is part of Rogers’ classic theory of Diffusion of Innovations (DOI): observability. Observability can be defined as the extent to which the results of using a technology are visible to others [44]. This is in line with the findings in the current study, in which we have also found that the use of technology by family members influenced the use of technology by older adults. However, STAM as described by Renaud and van Biljon, does not make explicit other types of social influence [48]. In addition to Renaud and van Biljon [48], Chen and Chan [49] have also proposed and tested a model, which they also call the Senior Technology Acceptance Model (STAM). The study by Chen and Chan [49] showed that satisfying and supportive personal relationships, as well as a high level of social activity, can have a positive effect on the self-reported use of various types of technologies by older adults. However, Chen and Chan [49] have not added or tested variables that can explain how and why personal relationships and social activity can affect technology use.

All in all, it seems that none of the abovementioned models captures all of our findings: the various ways in which family members influence technology use by older adults are scattered across various models. Moreover, none of the abovementioned models captures the underlying motivation of family members who influence technology use by older adults.

This paper was limited to the role of family members in technology acceptance of older adults. It should be noted that we asked our older respondents in an open way about the purchase and (intended) use of technology and factors that could be of influence. Although we did not explicitly ask about the role of spouses, children, or grandchildren, in most interviews, these roles naturally came to the fore, far more extensively than did the roles of other family members or peers. However, focusing explicitly on the role of family members is expected to provide additional valuable insights. Focusing on the role of the whole network is also important because the number of people growing old without children and grandchildren—or with children living at a greater distance—increases.

Furthermore, it should be noted that, in some cases, spouses were present during the interview. And although interviewers directed their questions to respondents, spouses sometimes clarified or added insights to the interview. This was helpful in understanding factors that influence technology acceptance, but it of course also influenced our results. However, to support a confidential environment for the interview, we do not think it is feasible to ask the spouse to leave the room during the interview.

While our study adds to the current literature by focusing on the behavior of older adults (*i.e.*, technology use) instead of just on their intention to use technology [17,50,51], it should be noted that we measured self-reported use. Research shows that studies based on self-reported use may show different results with studies employing direct usage measurement (*i.e.*, actual usage) [51,52]. This implies that the findings in our study cannot readily be compared with findings from studies that measure actual usage. Additionally, it cannot be ruled out that our self-reported (subjective) measurements of use are biased (*i.e.*, participants may have overestimated or underestimated their actual use) [52]. The current study indicates several directions for further research in addition to those mentioned before. To fully understand social dimensions of technology acceptance, it would be worthwhile to study the role of all members of the social network from the perspective of both older adults themselves and members of their social network. Such studies should focus on the social network in a broad sense, including peers, friends, but also professionals like general practitioners or caregivers and should focus on both motives and actions. Besides, it would be interesting to study the impact of members of the social network. Could a supportive network help to overcome difficulties in technology or help older adults to become more convinced of their own capacities? In addition, it would be of value to study the attitudes and opinions of various stakeholders (including older adults) involved in use of technology. This provides insight in how attitudes and opinions influence technology use. This is especially interesting when attitudes and opinions are conflicting between older adults and members of their social network.

## 5. Conclusions 

Our study reveals the importance of including family members when implementing technology in the lives of older adults. Because our study shows that parents are willing to try to use technology when their children are convinced of its positive effects, it is obvious that children should be provided with information about the value and use of devices to be implemented. Many children worry about their parents, so they will probably be ambassadors of certain technology when it helps to diminish their worries.

Our study also indicates that it could be of added value to determine an appropriate role of grandchildren when trying to stimulate technology acceptance by older adults. Grandchildren are both approachable and often skilled at working with technology, even at a (very) young age. Furthermore, older adults easily adopt their enthusiasm for technology; indeed, they are more willing to accept technology that their grandchildren like. This will not be the case for specifically designed assistive technology, but grandchildren can probably have a facilitating role when it comes to applications running on a regular tablet, smart phone, or other computer devices. Exploring the potential of the role of grandchildren for technology acceptance by older adults might also diminish the burden for children.
